# Data on Western blot and ELISA analysis of medaka (*Oryzias latipes*) follicle-stimulating hormone (Fsh) and luteinizing hormone (Lh) using recombinant proteins expressed with *Pichia pastoris*

**DOI:** 10.1016/j.dib.2019.01.034

**Published:** 2019-01-19

**Authors:** Susann Burow, Romain Fontaine, Kristine von Krogh, Ian Mayer, Rasoul Nourizadeh-Lillabadi, Lian Hollander-Cohen, Yaron Cohen, Michal Shpilman, Berta Levavi-Sivan, Finn-Arne Weltzien

**Affiliations:** a Department of Basic Sciences and Aquatic Medicine, Faculty of Veterinary Medicine, Norwegian University of Life Sciences, 0454 Oslo, Norway; b Department of Production Animal Clinical Sciences, Faculty of Veterinary Medicine, Norwegian University of Life Sciences, 0454 Oslo, Norway; c Department of Animal Sciences, Faculty of Agriculture, Food and Environment, The Hebrew University, Rehovot 76100, Israel

**Keywords:** Enzyme-linked immunosorbent assay, Follicle-stimulating hormone, Luteinizing hormone, *Oryzias latipes*, Recombinant gonadotropins, Western blot analysis

## Abstract

The gonadotropins follicle-stimulating hormone (Fsh) and luteinizing hormone (Lh) play essential roles in vertebrate reproduction. This article presents data on molecular weight validation of recombinant medaka (*Oryzias latipes*) (md) gonadotropins Fshβ (mdFshβ), Lhβ (mdLhβ), Fshβα (mdFshβα), and Lhβα (mdLhβα) generated by *Pichia pastoris,* as well as data on a validation of produced antibodies against Fshβ and Lhβ by Western blot analysis. Furthermore, the article includes data on Fsh and Lh protein levels in male medaka pituitaries using recombinant mdFshβα and mdLhβα within enzyme-linked immunosorbent assays (ELISAs), in which protein amounts were analyzed related to body weight and age of the fish. This dataset is associated with the research article entitled “Medaka Follicle-stimulating hormone (Fsh) and Luteinizing hormone (Lh): Developmental profiles of pituitary protein and gene expression” (Burow et al., in press).

**Specifications table**

**Table:** 

Subject area	Biology
More specific subject area	Physiology, Neuroendocrinology
Type of data	Image (Western blot analysis), Graph (ELISA)
How data was acquired	Data for validation of recombinant proteins and antibodies were acquired through Western blot, data for protein levels were obtained through ELISA using microplate spectrophotometer.
Data format	Analyzed
Experimental factors	Prior to Western blot analysis, *N*-glycosidase F was used to produce deglycosylated proteins by hydrolyzing all types of *N*-glycan chains.
Experimental features	Validation of medaka recombinant proteins and antibodies, and generation of pituitary Fsh and Lh levels in male medaka was performed.
Data source location	Department of Basic Sciences and Aquatic Medicine, Faculty of Veterinary Medicine, Norwegian University of Life Sciences, 0454 Oslo, Norway.
Data accessibility	Data are presented in this article.
Related research article	Burow, S., Fontaine, R., von Krogh, K., Mayer, I., Nourizadeh-Lillabadi, R., Hollander-Cohen, L., Cohen, Y., Shpilman, M., Levavi-Sivan, B., Weltzien, F.A., Medaka Follicle-stimulating hormone (Fsh) and Luteinizing hormone (Lh): Developmental profiles of pituitary protein and gene expression levels, Gen. Comp. Endocrinol. (in press) [1].

**Value of the data**

• The establishment of competitive ELISAs using recombinant medaka gonadotropins to quantify the content of Fsh and Lh, for the first time, extends the accessibility of quantitative methods for medaka and enables advanced functional studies on gonadotropin physiology in fish.
• The generated ELISA data determining pituitary Fsh and Lh protein levels in male fish during development in this article represent valuable data and a tool for future studies, since investigations in male fish during puberty are quite limited until today.
• The data on Fsh and Lh protein levels in male medaka pituitaries using recombinant mdFshβα and mdLhβα reveal that body weight explains the variance in the dependent variable (gonadotropin) better compared to age of the fish for Fshβ and Lhβ. In addition, body weight is indicated to explain the variance in the dependent variable for Lhβ better compared to Fshβ.
• The generation of specific antibodies against medaka Fshβ and Lhβ presented here will be a valuable tool for future experiments on gonadotropins in medaka, an important model organism in biology.

## 1 Data

**Fig. 1:**
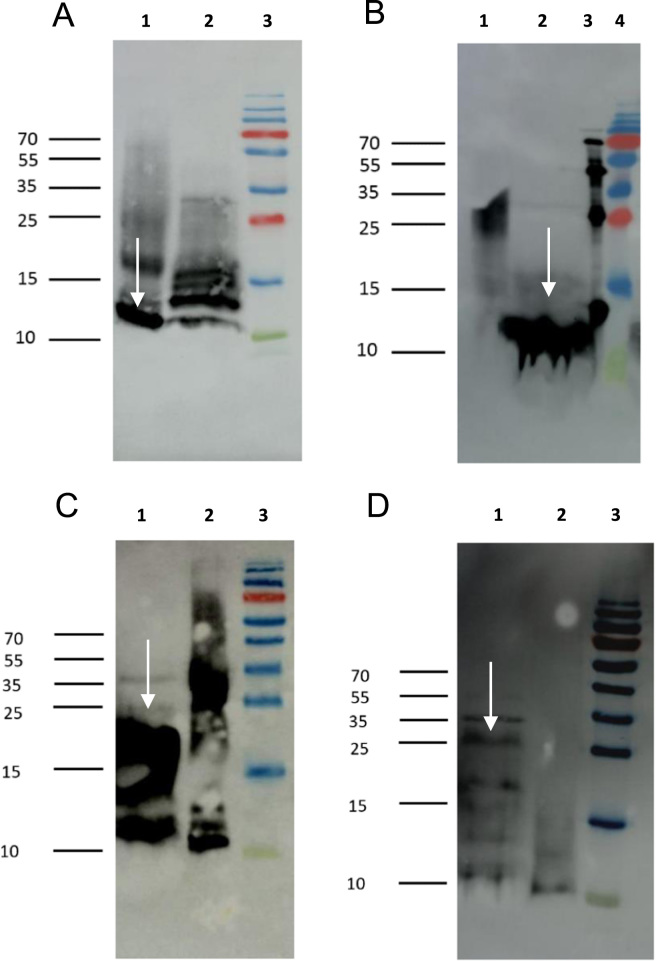
Molecular weight validation of mdFshβ, mdLhβ, mdFshβα, and mdLhβα expressed with *P. pastoris* by Western blot analysis. Supernatants of transformed *P. pastoris* cultures were separated by SDS-PAGE and immunoreacted with antibodies against His. PageRuler Plus Prestained Protein Ladder lane 3 (A), lane 4 (B), lane 3 (C), lane 3 (D). His-tagged Protein Standard lane 3 (B). The Western blot confirmed the expected molecular weight of A) mdFshβ, Lane 2 represents mdFshβ; Lane 1 represents deglycosylated mdFshβ. B) mdLhβ, Lane 1 represents mdLhβ; Lane 2 represents deglycosylated mdLhβ C) mdFshβα, Lane 2 represents mdFshβα; Lane 1 represents deglycosylated mdFshβα D) mdLhβα, Lane 2 represents mdLhβα; Lane 1 represents deglycosylated mdLhβα. White arrows indicate protein bands after deglycosylation with PNGase F.

The data on characterization of recombinant medaka (md) gonadotropins Fshβ (mdFshβ) (Fig. 1A), Lhβ (mdLhβ) (Fig. 1B), Fshβα (mdFshβα) (Fig. 1C), and Lhβα (mdLhβα) (Fig. 1D) by immunoreacting them against the His-tag demonstrated a clear validation since all recombinant proteins were successfully detected with His-tail antibodies, and their molecular sizes derived from Western blots were in accordance with the calculated estimates (according to sequence). Under reducing conditions, mdFshβ and mdFshβα were detected as bands of 14–16 kDa (Fig. 1A) and 25–30 kDa (Fig. 1C), respectively, and after deglycosylation with PNGase F as bands of 12–14 kDa (Fig. 1A) and 24–25 kDa (Fig. 1C), respectively. This is in accordance with the calculated molecular weight without glycosylation residues for mdFshβ (13 kDa) and for mdFshβα (25 kDa). Under reducing conditions, mdLhβ and mdLhβα had a molecular weight of 15 kDa (Fig. 1B) and 35 kDa (Fig. 1D), respectively, and after deglycosylation 12–14 kDa (Fig. 1B) and 27–28 kDa (Fig. 1D), respectively. Again, this was in accordance with the expectation for deglycosylated mdLhβ (15 kDa) and mdLhβα (28 kDa).

**Fig. 2:**
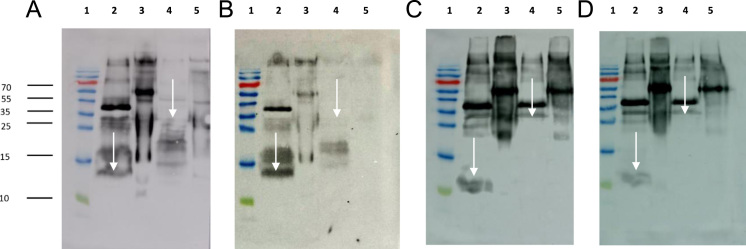
Validation of recombinant proteins mdFshβ, mdLhβ, mdFshβα, and mdLhβα expressed with *P. pastoris* by Western blot analysis. Supernatants of transformed *P. pastoris* cultures were separated by SDS-PAGE and immunoreacted with antibodies against mdFshβ (2A, 2B) and mdLhβ (2C, 2D). First lane represents PageRuler Plus Prestained Protein Ladder. The Western blot confirmed that the antibodies detected the correct proteins, and verified the absence of cross-reactions. A and B) mdFshβ and mdFshβα, Antibody against mdFshβ, Dilution 1:100.000 (2A) and 1:600.000 (2B); Lane 3 represents mdFshβ, lane 5 represents mdFshβα; Lanes 2 and 4 represent deglycosylated samples of those shown in lanes 3 and 5 respectively. C and D) mdLhβ and mdLhβα, Antibody against mdLhβ, Dilution 1:100.000 (2C) and 1:600.000 (2D); Lane 3 represents mdLhβ, lane 5 represents mdLhβα; Lanes 2 and 4 represent deglycosylated samples of those shown in lanes 3 and 5 respectively. White arrows indicate protein bands after deglycosylation with PNGase F.

Western blot analysis of antibodies produced against medaka Fshβ and Lhβ revealed specificity and absence of cross-reactions as all recombinant proteins mdFshβ, mdLhβ, mdFshβα, and mdLhβα were detected exclusively with antibodies against either medaka Fshβ (Fig. 2A, B) or Lhβ (Fig. 2C, D). Under reducing conditions and after deglycosylation, mdFshβ and mdFshβα were determined as bands of 12–13 kDa and 23–25 kDa (Fig. 2A, B), respectively. mdLhβ was revealed after deglycosylation very weakly as a band of 12–13 kDa, and mdLhβα was observed as a band of 27–29 kDa (Fig. 2C, D).

**Fig. 3:**
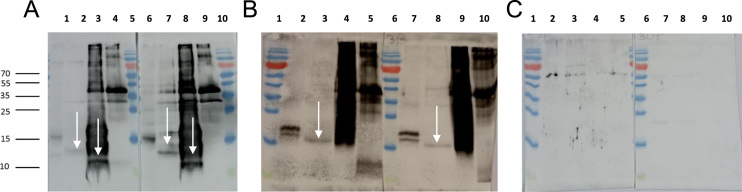
Validation of the produced antibodies against mdFshβ and mdLhβ and characterization of medaka pituitary extract, mdFshβ, and mdLhβ expressed with *P. pastoris* by Western blot analysis. Supernatants of transformed *P. pastoris* cultures were separated by SDS-PAGE and immunoreacted with antibodies against mdFshβ (3A) and mdLhβ (3B) and with medaka pre-immune serum (3C). The Western blot confirmed that the antibodies detected proteins of the right size in medaka pituitaries. A) Antibody against mdFshβ, Dilution 1:2000; Lane 1 (Rabbit 1 (R1)) and 6 (Rabbit 2 (R2)) represent medaka pituitary extract, lane 2 (R1) and 7 (R2) represent medaka pituitary extract after deglycosylation; Lanes 3 (R1) and 8 (R2) represent mdFshβ after deglycosylation; Lanes 4 (R1) and 9 (R2) represent deglycosylated samples of mdLhβ. Lane 5 and 10 represent PageRuler Plus Prestained Protein Ladder. B) Antibody against mdLhβ, Dilution 1:2000; Lane 2 (R1) and 7 (R2) represent medaka pituitary extract, lane 3 (R1) and 8 (R2) represent medaka pituitary extract after deglycosylation; Lanes 4 (R1) and 9 (R2) represent mdLhβ after deglycosylation; Lanes 5 (R1) and 10 (R2) represent deglycosylated samples of mdFshβ. Lane 1 and 6 represent PageRuler Plus Prestained Protein Ladder. C) The Western blot confirmed the validation of the produced antibodies and verified that the plasma taken before the final injections did not react with mdFshβ and mdLhβ. Medaka pituitary extract, mdFshβ, and mdLhβ were immunoreacted against medaka pre-immune serum as a negative control (test bleeding). Negative control: Pre-immune serum of Rabbit 1 (3C, lane 1 to 5) and Rabbit 2 (3C, lane 6 to 10); Lane 2 (R1) and 7 (R2) represent medaka pituitary extract, lane 3 (R1) and 8 (R2) represent medaka pituitary extract after deglycosylation; Lanes 4 (R1) and 9 (R2) represent mdFshβ after deglycosylation; Lanes 5 (R1) and 10 (R2) represent deglycosylated samples of mdLhβ. Lane 1 and 6 represent PageRuler Plus Prestained Protein Ladder. White arrows indicate protein bands after deglycosylation with PNGase F.

When using the antibodies on medaka pituitary extracts, native mdFshβ (Fig. 3A) and mdLhβ (Fig. 3B) could be detected. Using the mdFshβ antibody, bands of approximately 13 kDa were revealed for mdFshβ (Fig. 3A). When using the mdLhβ antibody, there was no clean band for mdLhβ due to very strong signals (Fig. 3B). No bands were revealed for mdLhβ with the mdFshβ antibody (Fig. 3A) and no bands for mdFshβ using the mdLhβ antibody (Fig. 3B). When medaka pituitary extract, recombinant mdFshβ, or recombinant mdLhβ were immunoreacted against rabbit pre-immune serum as a negative control (test bleeding), there was no specific band observed (Fig. 3C).

**Fig. 4:**
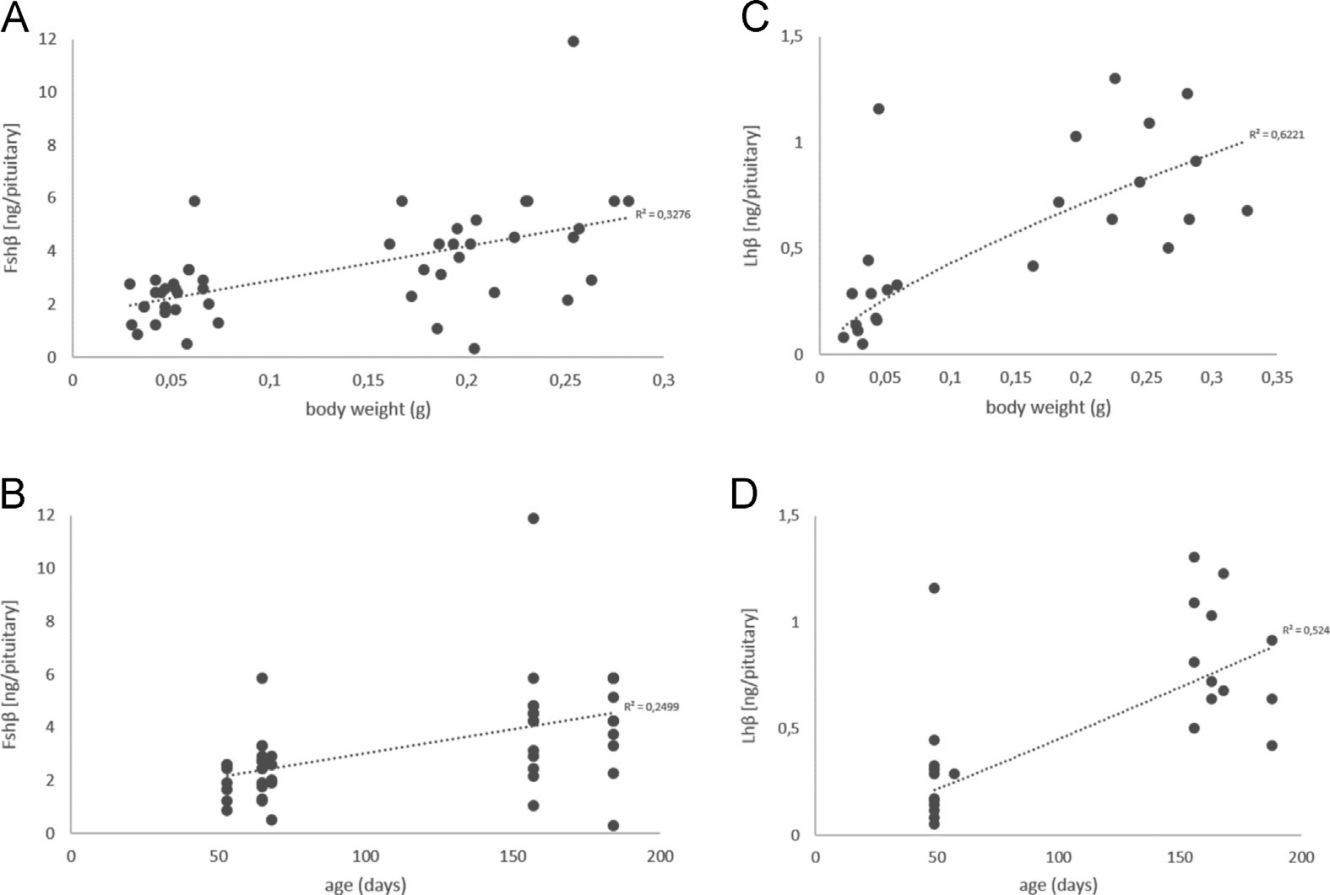
Profile of Fsh and Lh protein levels in pituitaries from juvenile and adult male medaka as a functional study obtained by ELISA. Quantified amounts of Fshβ (Fig. 4A, B) and Lhβ (Fig. 4C, D) (in ng/pituitary). Data have been analyzed as a function of body weight (Fsh Fig. 4A, linear trendline; Lh Fig. 4C, power trendline), and age of the fish (Fsh Fig. 4B, linear trendline; Lh Fig. 4D, power trendline). Body weight (*R*^*⁠*2^ = 0,3276; Fig. 4A) explains the variance in the dependent variable (gonadotropin) better compared to age of the fish (*R⁠*^2^ = 0,2499; Fig. 4B) for Fshβ using a linear trendline. As for Fshβ, body weight (*R*⁠^2^ = 0,6221; Fig. 4C) explains the variance in the dependent variable better compared to age (*R*⁠^2^ = 0,524; Fig. 4D) for Lhβ using a power trendline. Comparing the R^⁠2^ of body weight and age between the Fshβ and Lhβ profiles, it is important to note that the R⁠^2^s are higher for Lhβ than for Fshβ, indicating that body weight explains the variance in the dependent variable for Lhβ better compared to Fshβ.

Furthermore, this article provides data on Fsh and Lh protein levels in pituitaries from juvenile and adult male medaka that were obtained by enzyme-linked immunosorbent assay (ELISA). The data have been analyzed as a function of body weight (Fsh Fig. 4A, Lh Fig. 4C) and age of the fish (Fsh Fig. 4B, Lh Fig. 4D). Body weight (*R*^*⁠*2^ = 0,3276; Fig. 4A) explains the variance in the dependent variable (gonadotropin) better compared to age of the fish (*R*⁠^2^ = 0,2499; Fig. 4B) or body length (protein levels in relation to body length has been shown in Burow et al. [1]) for Fshβ using a linear trendline. As for Fshβ, body weight (*R*⁠^2^ = 0,6221; Fig. 4C) explains the variance in the dependent variable better compared to age (*R*^⁠2^ = 0,524; Fig. 4D) for Lhβ using a power trendline. Notably, the R⁠^2^s are higher for Lhβ than for Fshβ, indicating that body weight explains the variance in the dependent variable for Lhβ better compared to Fshβ. Since none of the R⁠^2^ is close to 1, a correlation of Fsh/Lh levels to either body weight or age of the fish is not indicated.

## 2 Experimental design, materials and methods

### 2.1 Animals

Japanese medaka (*Oryzias latipes*) of the dr-R strain were kept in re-circulating systems with light-dark cycle of L14:D10 and water temperature of 28 ± 1 °C. Embryos were incubated in embryo culture medium (E3; 5 mM NaCl, 0.17 mM KCl, 0.33 mM CaCl_2_, 0.33 mM MgSO_4_ (all Sigma-Aldrich, St. Louis, U.S.A.)), and kept at 26 °C until hatching and transfer to system tanks. The fish were fed three times per day with a combination of dry feed and live brine shrimp nauplii larvae (*Artemia salina*). Fish were raised under the same conditions with regard to temperature, photoperiod, food, tank size, and density. Handling, husbandry and use of fish were according to the guidelines and requirements for the care and welfare of research animals of the Norwegian Animal Health Authority and of the Norwegian University of Life Sciences. The work of the present article has been carried out in accordance with the EU Directive 2010/63/EU for animal experiments and Uniform Requirements for manuscripts submitted to Biomedical journals, and informed consent was obtained for experimentation with animal subjects.

### 2.2 Production and purification of recombinant gonadotropins mdFshβ, mdLhβ, mdFshβα, and mdLhβα, generation of specific antibodies for mdFshβ and mdLhβ, and Western blot analysis

Generation of recombinant proteins was conducted using the methylotrophic yeast *Pichia pastoris* (*P. pastoris*) expression system, generally according to Kasuto and Levavi-Sivan [2] and Yom-Din et al. [3], and described in detail in Burow et al. [1]. Synthesis of genes for medaka *fshb* (Accession Number NM_001309017.1), *lhb* (Accession Number AB541982.1), *fshba*, and *lhba* (*gpa*; Accession Number NM_001122906) was outsourced to GenScript, New Jersey, U.S.A. For each construct gene expression cassettes were generated with *P. pastoris* codon optimized DNA sequence. Polyclonal antisera against recombinant mdFshβ and mdLhβ were produced following a procedure according to Aizen et al. [4], which is reported in detail in Burow et al. [1].

For molecular weight validation, the purified recombinant proteins were analyzed by Western blot analysis using anti-His (diluted 1:2000), generally according to Yom-Din et al. [3]. To validate the produced antibodies, the recombinant proteins and medaka pituitary extract were visualized using anti-mdFshβ, or anti-mdLhβ (both diluted 1:2000, 1:100000, 1:600000) antisera. To confirm that the plasma of the rabbit before the final injections did not react with mdFshβ and mdLhβ, a Western blot using medaka pre-immune serum as a negative control against medaka pituitary extract, mdFshβ, and mdLhβ was performed.

### 2.3 Quantification of Fsh and Lh in male medaka pituitaries using ELISA

To quantify the content of Fsh and Lh protein levels in male medaka pituitaries, the ELISA methodology described in Burow et al. [1] was performed. For the profile of Fsh, pituitaries from 24 juvenile males with standard length (SL) between 12 mm and 16.5 mm, and of 24 adult males between 21 mm and 25.5 mm were used. Pituitaries from 12 juvenile males with SL between 12 mm and 16 mm, and of 12 adult males between 22.5 mm and 26.5 mm were dissected for the profile of Lh. For both Fsh and Lh 1 pituitary in 40 µl 0.1% BSA in PBST per biological replicate was used. Within the two groups juveniles and adults, body weight and age were measured, and protein amounts were analyzed related to body weight and age of the fish.

## Funding

This research was supported financially by the Norwegian University of Life Sciences and the Research Council of Norway (Grant number 248828 BioTek2021, and 231767 FriPro).
